# Supplementary material for: Application of machine learning in predicting consumer behavior and precision marketing
Source: PLoS One. 2025 May 6;20(5):e0321854. doi: 10.1371/journal.pone.0321854 (PMC12054852; doi:10.1371/journal.pone.0321854)
Supplement: Attachment — Submitted filename: Response to reviewers.docx [file pone.0321854.s002.docx]

Dear reviewers:

Thank you very much for reviewing our paper and your valuable suggestions. Your feedback is important for us to improve our work. We have carefully studied your comments and made detailed revisions to the paper based on your suggestions. Below are the responses to the specific questions and the description of the revisions.

Once again, we thank you for the time and effort you have put into our research and look forward to your further guidance!

Reviewer #1:

1. Originality
While some aspects show promise, it does not sufficiently demonstrate groundbreaking insights. The claims, while relevant, need further validation to establish originality and significance.
**Author response:** We recognize that although this study has made progress in predicting consumer behavior, it has not yet fully highlighted its originality. Therefore, in the introduction and conclusion, we further emphasize the innovation of this study, especially its advantages in processing large-scale and complex e-commerce data.

2. Technical Soundness
The manuscript employs specific methods or techniques, which seem appropriate on a surface level. However, the link between the data and conclusions lacks robustness. Some areas need clarification, especially in how findings directly support the stated outcomes.
**Author response:** We have further clarified how the data directly supports Model selection and conclusions in section 3.4 Model performance evaluation and comparison, and added a detailed comparison and discussion of the performance of individual models.

3. Main Claims and Significance
The primary claims potentially impactful, their significance for the discipline is moderate. The work but does not convincingly position itself as transformative within the field.
**Author response:** In the conclusion, we strengthen the paper's transformative discussion of precision marketing, especially its practical application and commercial value in the field of e-commerce.

4. Relationship to Literature
The paper demonstrates familiarity with existing literature but falls short in thoroughly contextualizing its contribution. These omissions detract from the paper's credibility and integration into the wider academic discourse.
**Author response:** We have strengthened the literature review in the introduction, and clearly pointed out the difference between this study and the existing literature, emphasizing its innovation.

5. Placement of Claims in Literature Context
The claims are not adequately framed within the context of existing literature. For instance, specific claim could be better supported by referencing to significant work. The discussion occasionally seems disconnected from the broader field.
**Author response:** We added additional citations to further compare the claims of this study with those in the existing literature.

6. Statistical Analysis
Statistical methods, including the analysis techniques, are employed but lack detailed explanation. The justification for the statistical choice is insufficient, and key assumptions are not thoroughly addressed. A more rigorous and transparent presentation of the analysis is needed to substantiate claims.
**Author response:** We have elaborated on the selection of statistical methods in section 3.4 Model performance evaluation and comparison, and explained how to evaluate model performance by multiple evaluation metrics (such as ROC AUC, accuracy, recall rate).

7. Potential for Resubmission
Despite its current shortcomings, the study has potential. Encouraging the authors to resubmit with revisions could elevate the work's quality and impact.
**Author response:** We have revised the paper based on the comments of the reviewers, especially in terms of data transparency, methodological clarity and literature integration, to ensure that the paper can better meet the publication criteria.

8. Data Availability
The paper does not clearly confirm that all underlying data are accessible. Transparent data sharing, including detailed datasets, would enhance the reproducibility and credibility of the findings.
**Author response:** We have added a data accessibility statement to the paper to ensure readers understand how to access research data.

9. Repository Information
No explicit mention is made of depositing data in public repositories. Providing accession numbers or repository links for specific data elements (e.g., genes, proteins, or other significant findings) is critical.
**Author response:** We have added a note about the data repository in the paper, providing specific information about the data storage.

10. Methodology
The methodology appears reasonably designed but lacks sufficient theoretical underpinning in some areas. Specific steps of methodology need clearer justification. Without this, reproducibility and confidence in the results are compromised.
**Author response:** We have added theoretical support for each method step in the methodological section and described each key step in the experimental process in more detail.
11. Reproducibility
Details of the methodology are insufficient to ensure reproducibility. Key parameters are omitted or underexplained.
**Author response:** We list all the important experimental parameters in detail in the methodological section and further explain the basis for their selection.

12. Results
The results section is descriptive but occasionally unclear. Graphs and tables, while helpful, would benefit from additional commentary to elucidate their connection to the hypotheses and conclusions.
**Author response:** We have added more notes and explanations to the results section to ensure that the meaning of each chart and table helps readers better understand the relationship to the hypotheses and conclusions.

13. Implications for Research, Practice, and Society
The paper briefly touches on its implications but does not fully explore their relevance to research, practice, or society. Expanding on these would strengthen its practical and theoretical contributions.
**Author response:** In the conclusion, we have expanded the implications of the paper for research, practice, and society, especially its implications for the e-commerce industry, precision marketing, and consumer behavior prediction.

14. Quality of Communication
The writing is technically accurate but lacks clarity in some areas. Sentence structure and jargon usage occasionally hinder accessibility. Simplifying language without sacrificing technical precision would improve communication.
**Author response:** We have simplified and rewritten some sentences in the paper, especially on technical terms and complex sentence structures, to improve readability and clarity.

15. Accessibility to Non-Specialists
The manuscript is primarily aimed at specialists, and non-specialists may find it difficult to engage with the content due to dense technical language and insufficient background context.
**Author response:** As suggested by the reviewers, we have simplified some technical terms and added more easy-to-understand explanations in the background section to ensure that non-specialist readers can understand the core content and applications of the research.

16. Standard English Usage
The manuscript generally adheres to standard English conventions but contains minor grammatical inconsistencies and awkward phrasing.
**Author response:** We have corrected grammatical inconsistencies and misphrasing in the paper and made sure that the sentences are more fluent.

17. Comments to the Author
• Ensure all data and methodologies are transparently presented to facilitate reproducibility.
• Address gaps in the literature review and align claims more closely with existing studies.
• Enhance clarity in the statistical analysis section, providing rationale and assumptions for methods used.
• Expand on the societal and practical implications of the research findings.

**Author response:** The paper demonstrates potential but requires substantial revision to meet publication standards. Key areas needing attention include data transparency, methodological clarity, and integration with existing literature. Encouraging a resubmission after thorough revision is recommended.

Reviewer #2:

 ABSTRACT

1. Ensure all abbreviations in the abstract, such as SVM, XGBoost, CatBoost, and BPANN, are clearly defined upon first use for better readability and comprehension.

support vector machine (SVM), extreme gradient boosting (XGBoost), categorical boosting (CatBoost), and backpropagation artificial neural network (BPANN)
2. Include performance metrics like F1-score etc. in the abstract for better clarity and impact.

Relevant performance metrics such as F1 scores have been added to the summary to enhance the clarity and impact of the summary and help readers better understand the performance of the model.
3. "Future research can further enhance the predictive power..." - This statement is **Author response:** overly generic and uninspiring. Specify actionable directions for future research, such as incorporating unstructured data (e.g., text, images) or experimenting with deep learning models like Transformers for consumer behavior analysis.
In the revision, actionable research directions are clearly proposed, such as the introduction of unstructured data (such as text, images, etc.), and attempts to use deep learning models (such as Transformers) for consumer behavior analysis to further improve the model's predictive ability and marketing effect.

INTRODUCTION:
1. Please format citations in square brackets (e.g., “[19]”) as per the submission guidelines.

**Author response:** We have followed the submission Guidelines by formatting all references in square brackets (e.g., "[19]") to ensure compliance with formatting specifications.

2. “Consumer behavior has become increasingly complex and unpredictable..." - This sentence reiterates what is already mentioned in the abstract without adding new information. Use this space to provide detailed background or evidence, such as specific challenges faced by industries.

**Author response:** We have revised this sentence and provided more detailed background and specific industry challenges, avoiding duplication and adding more meaningful background information.

3. "Traditional marketing models are unable to effectively cope with this change..." - This statement lacks support. Briefly explain why traditional models fail. For example, are they too rigid, or do they lack adaptability to real-time data?

**Author response:** We have added more specific notes that illustrate the limitations of traditional marketing models, especially when dealing with real-time data and dynamic change.

4. Does not provide sufficient evidence or citations to justify the selection of the machine learning models (SVM, XGBoost, CatBoost, BPANN) over other alternatives. Including references to studies or benchmarks demonstrating the superior performance, efficiency, or suitability of these models for consumer behavior prediction tasks would strengthen the argument.

**Author response:** We add more literature support in the introduction, citing research on the superiority and applicability of these machine learning models for consumer behavior prediction tasks.

5. The introduction fail to address potential limitations, such as computational complexity, data quality issues, or overfitting risks. Discussing these would strengthen the credibility of your research.

**Author response:** We have added a discussion of the potential limitations of the study in the introduction, particularly with regard to computational complexity, data quality issues, and the risk of overfitting.

6. Replace vague terms like "significant impact" and "great potential" with specific examples, data points, or case studies to ground your claims in reality.

**Author response:** Use specific cases and data to replace the original vague statements, so as to enhance the practical and persuasive argument.

7. The stated research objective, "to explore how to optimize consumer behavior prediction and precision marketing through machine learning," lacks originality and does not sufficiently highlight the study's unique contribution. To strengthen this section, clearly articulate the research gap.

**Author response:** The objective of the study is reformulated, the unique contribution of this study is clarified, and the research gaps in the current literature are described.

8. Please provide a suitable comparison with other state-of-the-art models to highlight the strengths and limitations of your approach in relation to existing methodologies.
**Author response:** The model used in this paper is compared with other cutting-edge models, and the advantages and limitations of our model in e-commerce data processing are illustrated.

Modeling Algorithms:
1. The section titled "Modeling Algorithms" should be renamed to "Methods and Materials" to align with standard academic conventions.

**Author response:** The title of the "Modeling Algorithms" section has been changed to "Methods and Materials" as recommended by the reviewers to comply with academic writing norms.

2. Additionally, within this section, include a detailed subsection that thoroughly explains the methodology of the study. This should cover how each machine learning model (SVM, XGBoost, CatBoost, BPANN) is implemented, the data collection, data preprocessing steps, feature selection, model architecture, model training and testing. performance metrics and any other relevant aspects of the experimental setup.

**Author response:** We have detailed the research methodology in the "Methods and Materials" section, covering data collection, pre-processing, feature selection, model architecture, training and testing procedures, and performance evaluation metrics. The implementation steps for each machine learning model are also described in detail.

3. Within the "Methods and Materials" section, for each machine learning algorithm used, provide a detailed explanation of why the selected model is the best choice for this study. Include supporting evidence from relevant literature or prior research that demonstrates the model's effectiveness in similar contexts.

**Author response:** We have further explained the reasons for the selection of each model in the "Methods and Materials" section, and cited relevant literature on the validity and applicability of these models in predicting consumer behavior.

4. For each machine learning algorithm used in the study, please include a visual representation (e.g., flowchart or diagram) of the algorithm’s implementation.
**Author response:** Based on the suggestions of reviewers, we have provided simple illustrations for some machine learning algorithms to help readers understand more clearly how each algorithm works and how it is implemented.

RESULTS AND DISCUSSION

1. The Results and Discussion section should focus on how the results relate to the hypothesis presented at the start of the study. It should provide a succinct explanation of the implications of the findings, particularly in relation to previous related studies. The detailed methodology, including Data Collection, Data Preprocessing, Feature Engineering, model training and OPtimization should be moved to the Methods and Materials section, as these describe the process rather than the outcomes. Please revise the manuscript to reflect this distinction.

**Author response:** The detailed methodology, including data collection, data preprocessing, feature engineering, model training, and optimization should be moved to the methods and materials sections.

2. Clarify the source of the dataset more explicitly. The mention of "UCI machine learning library" is sufficient, but you should also include the specific version or year to ensure reproducibility.

**Author response:** We have supplemented the specific source information of the dataset, including the dataset version in the UCI machine learning library, to ensure reproducibility of the results.

3. Please add a statistical metrics table (such as mean, standard deviation, etc.) to support the data analysis and provide a clearer summary of the key data characteristics.

**Author response:** Statistical characteristics of the dataset are not presented further at this time, but we will consider this in future studies and supplement these statistics where possible to help readers better understand the distribution and characteristics of the data.

4. Mention why SMOTE was chosen for addressing class imbalance and justify this choice with a brief explanation of its advantages.

**Author response:** We have clearly explained the rationale for choosing SMOTE (synthetic minority class oversampling technique) to solve the class imbalance problem and discussed its advantages.

5. It would be useful to explain why the threshold for variance filtering and mutual information was set to 0.05 and 0.05 respectively.

**Author response:** We have provided more explanations on the choice of setting the variance filtering threshold and the mutual information threshold to ensure that these parameters are set reasonably and based on evidence.

6. Clarify the performance metrics used (e.g., ROC AUC, accuracy, recall) and why they are important in assessing model performance.

**Author response:** We have further clarified the performance metrics used and explained their importance in evaluating model performance.

7. Consider discussing the trade-offs between model accuracy and model interpretability.

**Author response:** We include a discussion of the tradeoff between model accuracy and interpretability, especially for complex models such as XGBoost and CatBoost.

8. Include more details on the comparison metrics for the models.

**Author response:** Added more details for model comparisons, especially for comparison data on model performance.

9. Provide clearer interpretations of results in Table 2 and Figure 3.

**Author response:** We have explained the results in Table 2 and Figure 3 in more detail to ensure that the reader has a clear understanding of what each indicator and result means.

10. Add more context to the practical implications of feature importance analysis. How should companies act on these insights beyond the optimization suggestions?

**Author response:** Combined with feature importance analysis, we can implement strategies such as improving user engagement, personalized recommendation, reducing bounce rate and optimizing advertising.

11. The section on marketing strategy optimization seems theoretical. Provide examples or case studies where these strategies have been successfully implemented, or reference studies to support these suggestions.

**Author response:** We have added practical cases to the Marketing Strategy optimization section to help better illustrate how these strategies have been successfully implemented in practice and to provide relevant literature to support these recommendations.
12. Clarify the methodology used to optimize personalized recommendations, dynamic pricing, etc.

**Author response:** We have elaborated on ways to optimize marketing strategies such as personalized recommendations and provided specific steps on how to use machine learning and data analytics techniques to achieve these optimizations.

Conclusion and Prospect

1. The section can be renamed as "Conclusion and Future Scope".

**Author response:** We have changed the title of the "Conclusion and Prospect" section to "Conclusion and Future Scope" as suggested by the reviewers to more accurately reflect the content.

2. The conclusion appropriately summarizes the key findings but could benefit from clearer connections to the research questions and hypotheses stated earlier in the paper. Emphasize how the findings align with the initial objectives of the study.

**Author response:** We have revised the conclusions to emphasize the relationship between the findings and the original research objectives and assumptions to ensure that the conclusions are more clearly aligned with the research questions and objectives.

3. The suggestion to introduce additional data types such as social media behavior and geolocation is insightful but could be expanded. Explain how incorporating these data types could enhance model performance, and provide examples of their potential impact on prediction accuracy.

**Author response:** We have expanded our discussion on the introduction of unstructured data, such as social media behavior and geolocation data, explaining how these data types enhance model performance and providing examples of their potential impact on prediction accuracy.

4. The mention of combining deep learning or reinforcement learning is promising. However, it would be beneficial to elaborate on how these advanced techniques could be integrated into the current model framework, and what challenges might be encountered when doing so.

**Author response:** We have further elaborated on how to integrate deep learning and reinforcement learning techniques into existing model frameworks and discussed possible challenges such as computational complexity, data requirements, and training time.

5. The term "real-time decision optimization" could be clarified. It would be useful to specify which specific real-time applications, such as personalized recommendations, dynamic pricing, or user loyalty management, could benefit from the improved model.

**Author response:** The application of the improved model to practical scenarios such as personalized recommendation, dynamic pricing and user loyalty management is explained.

6. The future scope mentions incorporating emerging technologies but should also emphasize how future work could address the limitations or gaps identified in the current study. This could include areas for refinement in model performance or alternative strategies to overcome challenges observed.

**Author response:** We have further highlighted how future studies will address the limitations of current studies and proposed strategies to improve model performance and overcome challenges.

Reviewer #3:

1- In marketing we need to study about 4Ps at least ....what about product and placing data ? have you consider such a parameters in your modeling ?

**Author response:** We do take into account features related to "product" and "placement" in our models, especially when making market segmentation and personalized recommendations. These factors have an important impact on consumer behavior, especially in the e-commerce platform, product characteristics (such as category, price) and placement characteristics (such as product recommendation location, display page, etc.) directly affect the user's purchase decision. We take these features as important inputs and make appropriate processing and optimization in the feature engineering process.

2-Authers must put data collection as section two and make the results and discussion section four and methodology as a section three.

**Author response:** We have adjusted the structure as suggested by the reviewers, moving the "Data collection" section to Part 2, the "Methods" section to Part 3, and merging the "Results and Discussions" into Part 4 to conform to the standard academic structure.

3-The literature review is very limited and need to be extended to include several other researches published in 2023-2024.

**Author response:** We have expanded the literature review to include relevant studies from 2023-2024, providing more comprehensive background information that further supports the theoretical basis of our study.

4-The methodology modeling flowchart is not presented and need to be added for the readers benefits.

**Author response:** Describe the overall process of modeling more clearly through text.

5-The discussion is very shallow and need to be extended with other feature dimensions.

**Author response:** We have expanded the discussion section to include more discussion of feature dimensions, including how different features affect the prediction in different models, and how to further optimize feature selection.

Reviewer #4:

The manuscript titled “Application of Machine Learning in Predicting Consumer Behavior and Precision Marketing” addresses a highly relevant topic for today’s digital economy. The comparative evaluation of machine learning models—SVM, XGBoost, CatBoost, and BPANN—adds value by providing insights into consumer behavior prediction. The study’s feature importance analysis and its practical suggestions, such as personalized recommendations and dynamic pricing, are particularly noteworthy. That said, there are areas where the paper could be improved to enhance clarity, depth, and overall impact.

Clarify the research gap and intent in the introduction. While the introduction highlights the broader issues of consumer behavior and precision marketing, it does not clearly identify the specific research gap this study addresses. What problem in consumer behavior prediction does this work solve that others have not? Additionally, the paper needs a sharper focus on its novelty. For instance, what makes this comparison of models unique in the current landscape of machine learning applications? Explicitly stating these elements will position the study more clearly and strengthen its contribution.
**Author response:** We have clarified the research gaps and research intentions, and elaborated on the specific problems that this study solves in the prediction of consumer behavior, especially the processing ability of complex data in the e-commerce environment. The innovation of this study is reflected in the comparison of four models, CatBoost, XGBoost, SVM and BPANN, which fills the gap in the application comparison of these models on e-commerce platforms in the current literature.

Add a dedicated literature review section. Currently, there is no structured discussion of prior research, which makes it difficult to understand the groundwork that led to this study. A literature review should summarize recent advancements in machine learning for marketing, highlight key works, and point out gaps that justify the need for this research. Make sure to include references to recent studies from 2023 and 2024, as the field of machine learning and marketing is evolving rapidly. This will not only make the paper more current but also situate it within the broader academic conversation.
**Author response:** We have added a literature review section that reviews the relevant research in machine learning and precision marketing in recent years, focusing on the latest progress in 2023-2024, and illustrating the relationship between this study and existing research.

Include a clear methodology section before modeling. The paper jumps into describing the models without providing a proper methods section. This section should outline the research design, the choice of models, and why these specific techniques (SVM, XGBoost, CatBoost, BPANN) were chosen over alternatives like LightGBM or Random Forest. Additionally, elaborate on preprocessing decisions, such as feature selection, handling imbalanced data with SMOTE, and outlier removal. Providing detailed justifications here will improve the paper’s reproducibility and transparency.
**Author response:** We have reorganized the paper so that the methodological section now precedes the model description, and detail the study design, rationale for model selection, and data preprocessing (e.g. SMOTE, outlier removal, etc.).
Develop a discussion section to interpret the results. The results section does a good job of presenting the model performance and feature importance analysis, but it lacks a deeper discussion. Explain why CatBoost and XGBoost outperformed other models—what aspects of these techniques make them more suitable for handling complex e-commerce data? Also, compare your findings with similar works done in this area to highlight alignment or differences. To make this section more engaging for practitioners, consider incorporating feedback from real-life e-commerce marketers to frame the results in terms of their business impact, such as ROI improvement or customer segmentation strategies.
**Author response:** In the discussion section, we take a closer look at why CatBoost and XGBoost are better than other models and explore their advantages when dealing with complex e-commerce data. At the same time, the comparison with existing research is added, the advantages and limitations of CatBoost and XGBoost are discussed, and more practical insights are provided in conjunction with real e-commerce business feedback.

Add a limitations and implications section. It’s important to acknowledge the limitations of the study, such as the use of a static dataset that may not capture the dynamic nature of consumer behavior. Discuss how this could affect the results and what steps can be taken to address it in future research. On the implications side, connect the findings to practical applications—how can businesses use these models to improve marketing outcomes? This section would be particularly valuable for bridging the gap between research and industry practice.
**Author response:** We added the research limitations and practical significance sections, pointing out the limitations of this study, such as the use of static data sets that failed to fully reflect dynamic consumer behavior. At the same time, we also provide future research directions, including how to further improve model performance by introducing real-time data or more diverse data sources.

Conclude with a strong conclusion section. The paper ends abruptly without summarizing the key findings or outlining future directions. A clear conclusion should highlight the main contributions of the study, such as the performance of CatBoost and XGBoost, and their potential for e-commerce applications. It should also suggest future work, like exploring deep learning approaches, real-time data analysis, or expanding the dataset to include other industries and consumer segments.
**Author response:** In the conclusion, we summarize the key findings of the study and suggest future research directions.

Consider integrating a theoretical framework. The study would benefit from being grounded in a marketing or behavioral theory to connect the findings to a broader context. For example, frameworks like Behavioral Economics (decision-making) or Customer Relationship Management (CRM) could add depth to the study. This would elevate the paper’s academic value and provide readers with a stronger conceptual understanding of the work.
**Author response:** we mentioned that future studies can apply theoretical frameworks such as social media behavioral data or financial healthcare to model optimization to further enhance theoretical depth.

Minor refinements for clarity and engagement. While the paper is generally clear, there are areas where the writing could be polished for better flow and readability. For instance, some figures and tables could benefit from more descriptive captions to help readers quickly understand the takeaways. Additionally, improving the grammar and refining terminology (e.g., "administrative pages") would make the paper more professional and accessible.
**Author response:** We have optimized the language and terminology to ensure that the expression is more fluid and easy to understand. Changes have also been made to the title and description of the chart to make it clearer.

In summary, this paper tackles a critical and timely issue in precision marketing and demonstrates the value of machine learning models in predicting consumer behavior. The comparative analysis and practical insights are strengths of the study. To further improve its quality, I recommend clarifying the research gap, adding a literature review and methodology section, developing a robust discussion, and including practical business perspectives. With these refinements, the paper will be far more impactful for both academic researchers and industry professionals
